# Load Distribution in the Lumbar Spine During Modeled Compression Depends on Lordosis

**DOI:** 10.3389/fbioe.2021.661258

**Published:** 2021-06-10

**Authors:** Andreas Müller, Robert Rockenfeller, Nicolas Damm, Michael Kosterhon, Sven R. Kantelhardt, Ameet K. Aiyangar, Karin Gruber

**Affiliations:** ^1^Institute for Medical Engineering and Information Processing (MTI Mittelrhein), University Koblenz-Landau, Koblenz, Germany; ^2^Mechanical Systems Engineering, Swiss Federal Laboratories for Materials Science and Technology (EMPA), Duebendorf, Switzerland; ^3^Department of Mathematics and Natural Sciences, Institute of Sports Science, University Koblenz-Landau, Koblenz, Germany; ^4^Department of Mathematics and Natural Sciences, Mathematical Institute, UniversityKoblenz-Landau, Koblenz, Germany; ^5^Department of Neurosurgery, University Medical Centre, Johannes Gutenberg-University Mainz, Mainz, Germany; ^6^Department of Orthopedic Surgery, University of Pittsburgh, Pittsburgh, PA, United States

**Keywords:** biomechanics, forward dynamics, MBS model, musculo skeletal model, lumbar lordosis, curvature, Cobb angle

## Abstract

Excessive or incorrect loading of lumbar spinal structures is commonly assumed as one of the factors to accelerate degenerative processes, which may lead to lower back pain. Accordingly, the mechanics of the spine under medical conditions, such as scoliosis or spondylolisthesis, is well-investigated. Treatments via both conventional therapy and surgical methods alike aim at restoring a “healthy” (or at least pain-free) load distribution. Yet, surprisingly little is known about the inter-subject variability of load bearings within a “healthy” lumbar spine. Hence, we utilized computer tomography data from 28 trauma-room patients, whose lumbar spines showed no visible sign of degeneration, to construct simplified multi-body simulation models. The subject-specific geometries, measured by the corresponding lumbar lordosis (LL) between the endplates of vertebra L1 and the sacrum, served as ceteris paribus condition in a standardized forward dynamic compression procedure. Further, the influence of stimulating muscles from the *M. multifidus* group was assessed. For the range of available LL from 28 to 66°, changes in compressive and shear forces, bending moments, as well as facet joint forces between adjacent vertebrae were calculated. While compressive forces tended to decrease with increasing LL, facet forces were tendentiously increasing. Shear forces decreased between more cranial vertebrae and increased between more caudal ones, while bending moments remained constant. Our results suggest that there exist significant, LL-dependent variations in the loading of “healthy” spinal structures, which should be considered when striving for individually appropriate therapeutic measures.

## 1. Introduction

The spine constitutes a highly mobile skeletal structure with a wide inter-individual variation in the characteristics of its double-S shape. High mechanical stresses in daily life and sports may cause injuries that trigger long-term degenerative processes of the intervertebral disks (IVD) or the facet joints. The lumbar spine is particularly affected by degenerative phenomena because it carries the whole weight of the body above the affected level (Hajihosseinali et al., 2015). Deviations in the double-S shape may alter internal load distributions and accelerate degenerative processes. These deviations are commonly quantified using the *Cobb method* (Cobb, 1948), which was originally introduced to describe degrees of scoliosis, i.e., deviations in the coronal plane (White and Panjabi, 1990, Chapter 3.1). Adapting the Cobb method to the sagittal plane, the *lumbar lordosis* (LL) can be defined as the sagittal Cobb angle between upper endplate of vertebra L1 and the endplate of the sacrum (SA). In contrast to scoliosis, quantitative investigations regarding the effects of deviations in the sagittal curvature on spinal load distributions are scarce, especially when distinguishing between thoracic kyphosis (Briggs et al., 2007; Bruno et al., 2012) and lumbar lordosis (Keller et al., 2005; Bruno et al., 2017). While the latter studies were principally able to show an effect of changes in lordosis on the load distribution within the lumbar spine, a depiction of quantitative dependencies is to date still missing.

Understanding the variability in loading of certain spinal structures can be beneficial in clinical contexts, e.g.,for the classification of pathologies or planning of surgical interventions. It is assumed that degenerative alterations are a result of sagittal imbalance (Glassman et al., 2005), which can be measured, for example, by the sagittal vertical axis, i.e., the minimal distance between the C7 plumb-line and the posterior-superior vertebral corner of SA (Jackson and McManus, 1994), or the odontoid hip axis, i.e., the angle between the vertical line through the hip axis and a line from the hip axis to the dens of C2 (Le Huec et al., 2019). A further important characteristic constitutes the *spino-pelvic configuration*, usually represented by the three angular measurands pelvic incidence (PI), sacral slope (SS), and pelvic tilt (PT). It holds PI = SS+PT and it is assumed that optimal sagittal balance corresponds to a small PI-to-LL difference (ΔPILL), particularly |ΔPILL| = |PI−LL| ≤ 15° (Rothenfluh et al., 2015). Higher discrepancy between these two parameters is thought to result in spinal diseases and malfunctions (Roussouly and Pinheiro-Franco, 2011; Senteler et al., 2014; Bassani et al., 2019). For example, hyperlordosis (large LL) is assumed to accelerate discopathies and facet joint degenerations, whereas hypolordosis (small LL) is connected with high compressive peak forces in the IVDs. These and similar plausibility statements frequently occur in the literature, however, mainly in absence of a corresponding quantification (cf. Shirazi-Adl et al., 2002; Keller et al., 2005; Meakin et al., 2009; Gezelbash et al., 2016; Jentzsch et al., 2017). Hence, the aim of this study was to conduct a quantitative investigation regarding the influence of varying LL on the load distribution within the lumbar spine using forward dynamic models.

## 2. Model and Methods

**Figure 1:**
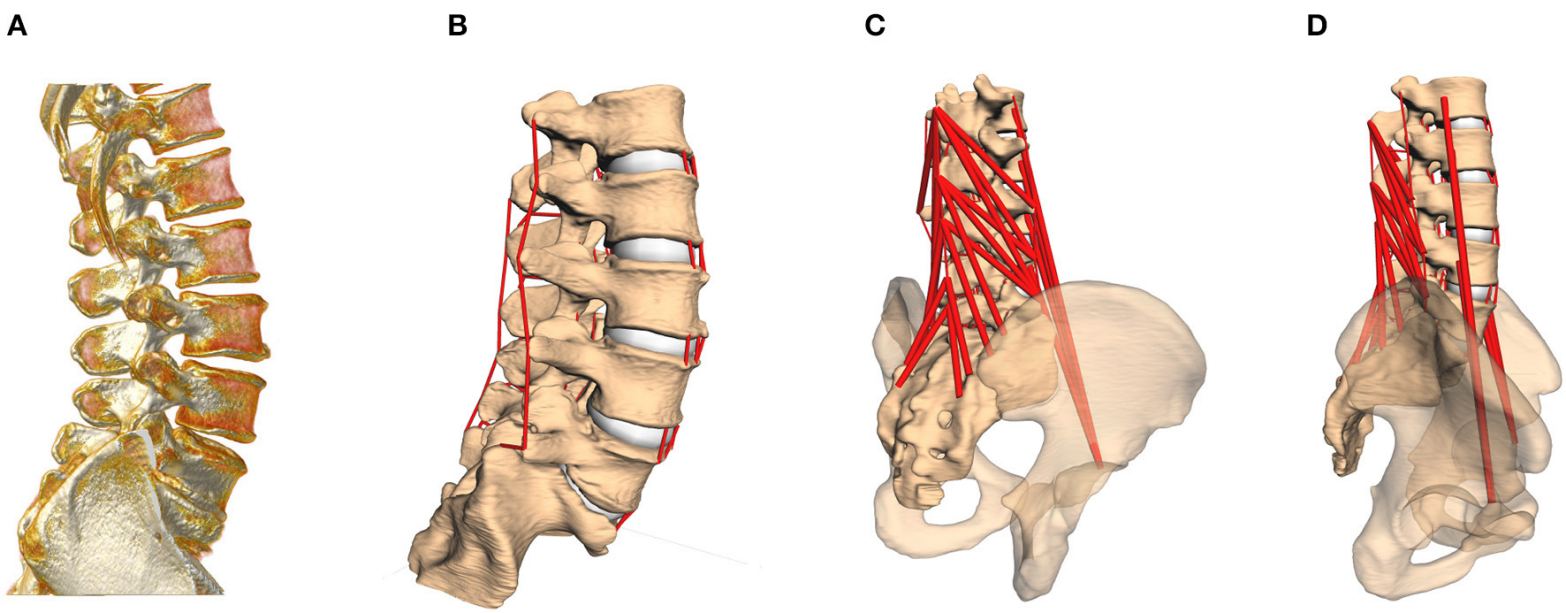
**(A)** CT image of the lumbar spine, rotated to standing position. **(B)** Computer model based on the subject-specific CT geometries, including passive structures [intervertebral disks (IVDs), facet joints and ligaments]. **(C,D)** In a last step, active force elements, muscles, are inserted into the model according to individual landmarks. The pelvis serves as origin for the *M. psoas major* group.

A total of 28 lumbar spinal models were constructed on the basis of *in vivo* computer tomography (CT) data from trauma-room patients with otherwise healthy spines (i.e., no signs of degeneration; 32.7 ± 14.5 years, where the age of two subjects was not known), provided anonymized by the University Medical Center in Mainz (Figure 1A). These images were taken in supine position, where the loading of spinal structures is significantly reduced compared to standing position (Wilke et al., 1999, Table 1). After semi-automatic segmentation, the resulting surfaces were loaded as rigid bodies into the MBS tool Simpack (Dassault Systèmes Deutschland GmbH, Munich, Germany), and oriented to upright (standing) position, under preservation of the subject-specific geometries, namely curvature, disk space, and facet joint gap. The whole lumbar spine was rigidly re-oriented from supine to upright without altering the relative, intersegmental orientations. “Upright” was defined such that the cranial endplate of the L3 vertebra was oriented parallel to the transversal plane (Rupp et al., 2015, Table 1), i.e., perpendicular to the line of action of the gravitational force. The individual L3 vertebral orientation with respect to the other vertebrae remained unchanged from the original supine state. This definition was maintained for all the models to ensure comparability. The model details described in the following had been previously validated against *in vitro* and *in vivo* data (Damm et al., 2019).

The six degrees-of-freedom, visco-elastic intervertebral body joints between two adjacent vertebrae, representing IVDs, were modeled by non-linear torque-angle and compressive force-deformation characteristics as well as linear shear force-deformation and damping (Damm et al., 2019, Figure 4, Equations 1 and 2). The center of mass of the sacrum was placed in the origin of a coordinate system, where positive *z* points upwards (cranial), positive *y* points frontal (anterior), and positive *x* points right (dexter). As compressive force was measured as the vertical (superior–inferior) part of the force in each reference frame, it could also be referred to as *z*-force. Accordingly, the (anterior–posterior) shear force is referred to as *y*-force and the flexion-extension moment around the transversal axis as *x*-torque. Facet joints were represented by one degree-of-freedom, linear visco-elastic force elements, oriented perpendicular to the regression plane between the (curved) surfaces of the adjacent superior and inferior articular facets. Forces were measured perpendicular to the regression plane between the superior and inferior articular facets, where negative (pulling) forces were not considered for these structures. In fact in some cases, particularly for the upper spinal levels, the facet force remained at 0 N, indicating the absence of compression, i.e., no facet surface contact due to the loading distribution. Instead, the capsule ligaments were compensating for the pulling force.

Next, subject-specific ligament and muscle insertion points on the bony surfaces were identified by anatomical landmarks (Schünke et al., 2015), checked and confirmed by the clinical co-authors (neuro-surgeons from the University Medical Center in Mainz), and connected by one-dimensional force elements (see Figures 1B–D). Ligaments were likewise modeled as visco-elastic passive elements, exhibiting a non-linear force-lengths characteristic and linear damping (Damm et al., 2019, Figure 5, Equations 6 and 7). With regard to muscle representation, *M. multifidus* and *M. psoas* major were modeled by point-to-point Hill-type active force elements (Rockenfeller and Günther, 2016, Appendix A). For both ligaments and muscles, pre-strain, and slack lengths, respectively, were scaled with the subject-specific geometries (Rockenfeller et al., 2020). Maximum muscle forces were adapted from the literature (Christophy et al., 2012), with 21 N for strands from the *M. multifidus* and 80 N for strands from the *M. psoas major*.

**Figure 2:**
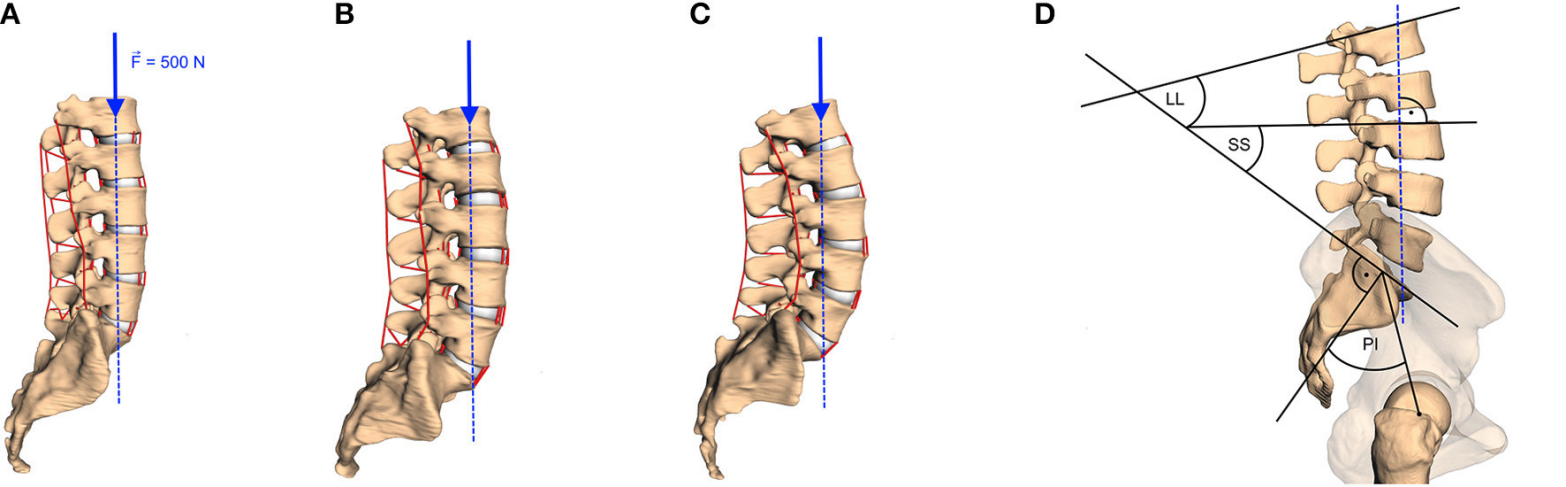
Examples of lumbar spinal curvature: **(A)** hypolordotic (LL = 28°), **(B)** regular (LL = 49.2°), and **(C)** hyperlordotic (LL = 66.3°). The method of calculating the LL, the sacral slope (SS), and the pelvic incident (PI) is sketched in **(D)** and described in the text. A vertical force of 500 N (blue arrows with dashed line of action) was applied on the COM of the vertebral body of L1 in all models.

To ensure maximum possible comparability between our forward dynamic loading simulations, boundary conditions for each spine were standardized as follows: First, the lumber spine was encastered at the sacrum level. Second, the anterior–posterior and medial-lateral translation of vertebra L1 was prohibited to avoid tilting, while the other degrees of freedom were not restricted. This restriction was supposed to represent multi-level stabilizing musculature, which was not implemented here, in order to emphasize the effect of changes in load distribution per change in LL. Third, a load of 500 N, representing the upper body weight (Nachemson, 1981, Table 1), was applied on the center of mass of the vertebral body L1 (see Figure 2). Fourth, a standardized forward dynamics simulation of a 2 s time horizon, ensured each spine to reach a final equilibrium state

Fourth, possible influence of muscle activity on spinal loading was investigated for only the *M. multifidus* group, which is known to have a stabilizing effect on the lumbar spine (Macintosh and Bogduk, 1986; Danneels et al., 2001; Ward et al., 2009). Therefore, a total of five different scenarios regarding muscle participation were conducted: (i) “no muscles,” denoting the absence of any active or passive muscle force, (ii) “passive muscles (*u* = 0),” denoting the absence of any neural stimulation/excitation 0 ≤ *u* ≤ 1 (cf. Rockenfeller and Günther, 2016), and (iii)–(v) “active muscles (*u* = 0.1, 0.25, or 0.5),” denoting the degree of stimulation of the *M. multifidus* group.

**Figure 3:**
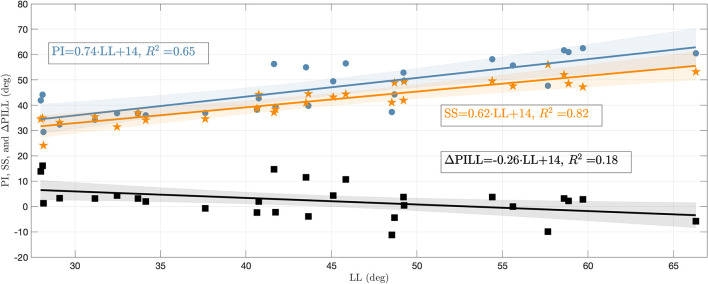
PI, SS, and ΔPILL plotted against LL. The blue circles represent the PI, orange asterisks the SS, and black squares the ΔPILL for the 28 individual lumbar spines. Regressions lines (with confidence bands) are displayed in corresponding colors and their equations as well as coefficients of determination (*R*^2^) are stated in the annotations.

To assess the influence of the sagittal curvature on the simulation results, we defined the LL as a measure parameter to define the degree of lumbar lordosis (Vrtovec et al., 2009). Therefore, the cranial endplates of L1 and SA are virtually extended and their intersection angle in the sagittal plane is determined, cf. Figure 2D. From the available data, we obtained a mean LL of 44.0 ± 11.0° with a range between 28.0 and 66.3°, which corresponds well with literature data (Chernukha et al., 1998; Lafage et al., 2009). Smaller LL indicate hypolordotic spines (Figure 2A) and larger LL indicate hyperlordotic spines (Figure 2C). However, it should be noted that the LL alone does not necessarily constitute a unique measure, as different internal (L2–L5) curvatures may correspond to the same overall LL (Been and Kalichman, 2014, Figure 2). Therefore, we additionally compared the LL to the anatomic parameters that characterize the *sagittal balance*, namely PI, SS, and ΔPILL. The PI is the angle between the lines going from the midpoint of the line connecting the femur heads to the midpoint of the S1 endplate and the normal of the S1 endplate at this midpoint. The SS is the angle between the S1 endplate and the transversal plane (Lafage et al., 2009) (see again Figure 2D). The ΔPILL value is the difference between PI and LL. We obtained a PI of 46.3 ± 10.1° (mean ± standard deviation) with a range between 29.5 and 62.5°, a SS of 41.7 ± 7.6° with a range between 24.1 and 56.0°, and a ΔPILL of 2.3 ± 6.6° with a range between −11.2 and 16.0°. Figure 3 shows the relation between LL and PI (*R*^2^ = 0.65), LL and SS (*R*^2^ = 0.82), as well as LL and ΔPILL (*R*^2^ = 0.18) for our 28 samples. On average, an increase of one degree LL was associated with an increase of ~0.74° in PI and 0.62° in SS, which well corresponds to literature data—cf. Roussouly et al. (2005, Tables 1, 2) and Naserkhaki et al. (2016, Figure 1). For ΔPILL, we found a decrease of 0.26° per degree LL, which has, to our knowledge, not yet been reported. As only a single hypolordotic spine exhibited a |ΔPILL|>15°, we did not perform ΔPILL-dependent analysis, as presented in Senteler et al. (2014); Rothenfluh et al. (2015).

The output quantities, which were assumed to depend on the LL, obtained from our standardized forward dynamic simulations, were (i) the changes in sagittal Cobb angles for all vertebrae between the start (*t* = 0s, no loading) and the end (*t* = 2s, loaded equilibrium) of the simulation, (ii) the compressive (*z*-)forces in the IVDs between two adjacent vertebrae, (iii) the IVD shear (*y*-)forces, (iv) the IVD torques around the transversal (*x*-)axis, and (v) the facet joint forces. To assess the LL dependency of these quantities, a regression line for each vertebra (respectively level) and for each mode was calculated in a least-squares sense. A subsequent significance-of-correlation *t*-test was carried out, using the test statistic

$$\begin{array}{l} T=\frac{r\cdot \sqrt{n-2}}{\sqrt{1-r^{2}}}, \end{array}$$

where *r* = cor(LL, *Y*) denotes Pearson's correlation coefficient between LL and the observed quantity *Y*, and *n* = 27 (number of available spinal models minus one) the degrees of freedom (see Zar, 1972). The corresponding *p*-value was calculated as

$$\begin{array}{l} p=2\cdot \left(1-F_{t_{n}}\left(T\right)\right), \end{array}$$

where *F*_*t*_*n*__ denotes the cumulative distribution function of the Student's *t*-distribution with *n* degrees of freedom. Small *p*-values indicate that the observed correlation is unlikely under the null hypothesis “*r* = 0,” which should thus be rejected. All obtained correlations *r* along with the corresponding *p*-values are summarized in Table 1 (Appendix A). The resultant slopes *s* of the regression lines can be calculated by *s* = *r* · σ(*Y*)/σ(LL), with σ being the standard deviation operator. Uncertainty of the regression analysis is indicated by 95% confidence bands *f*(LL) ± ω(LL) around the regression line *f*(LL), with

$$\begin{array}{l} \omega \left(\text{LL}\right)=t_{n-2,0.95}\cdot \sigma \left(Y\right)\cdot \sqrt{\frac{1}{n}+\frac{{\left(\text{LL}-\bar{\text{LL}}\right)}^{2}}{{\left(n-1\right)}^{2}\cdot \sigma {\left(LL\right)}^{2}}}, \end{array}$$

where *t*_*n*−2,0.95_ denotes the 95%-quantile of the *t*-distribution with *n*−2 degrees of freedom, and $\bar{\text{LL}}$ the mean value of LL. Slopes for all modes and output quantities, together with their 95% confidence intervals (CI), are listed in Table 2 (likewise Appendix A).

## 3. Results

**Figure 4:**
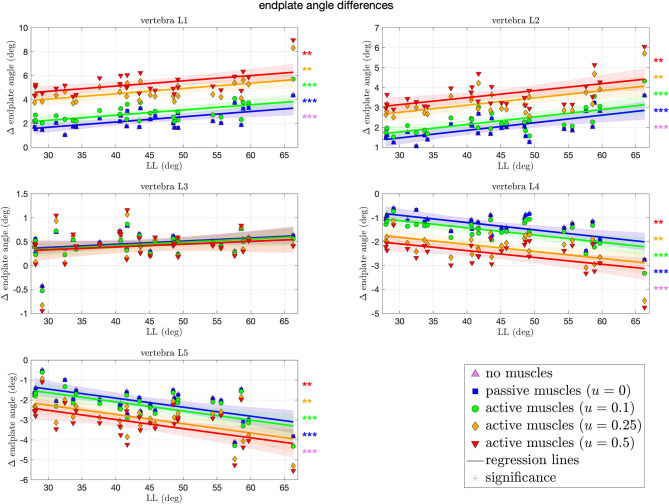
Differences in Cobb angles during compression of all 28 vertebrae against LL. Colors and marker symbols of the data points and the corresponding regression lines consistently correspond to the modes: lilac up-pointing triangles for simulations without muscles involved; blue squares for passive muscles; as well as green circles, orange diamonds, and red down-pointing triangles for muscle stimulation of *u* ∈ {0.1, 0.25, 0.5}, respectively. Confidence bands of the regression are shown as pale areas of the corresponding color. The significance of the statistical test is indicated by alongside asterisks (** = significant with 0.001 ≤ *p* < 0.05, *** = highly significant with *p* < 0.001).

Orienting the spine upright from supine position, as well as applying loading and possibly muscle forces, changes the initial LL between start (*t* = 0 s) and end (*t* = 2s) of the simulation. In Figure 4, these changes are quantified for each spinal level and each muscle stimulation protocol. Expectedly, for the mid vertebra L3, no significant changes in the Cobb angle were observed for any muscle stimulation. For the neighboring vertebrae L2 and L4, we observed a moderate, yet (highly) significant, increase, and decrease, respectively, of ~1.5° over the whole LL range, i.e., ~0.03° change per degree LL (see Table 2 in Appendix A for concrete values and CI). This trend is continued for the outer vertebrae L1 and L5, where a higher change of ~2° (0.045° per degree LL) increase for L1 and decrease for L5 is observed over the whole LL range. Notably, changes in scenarios with highly stimulated muscles were less significant than for passive or moderately stimulated muscles, indicating a stabilizing effect.

**Figure 5:**
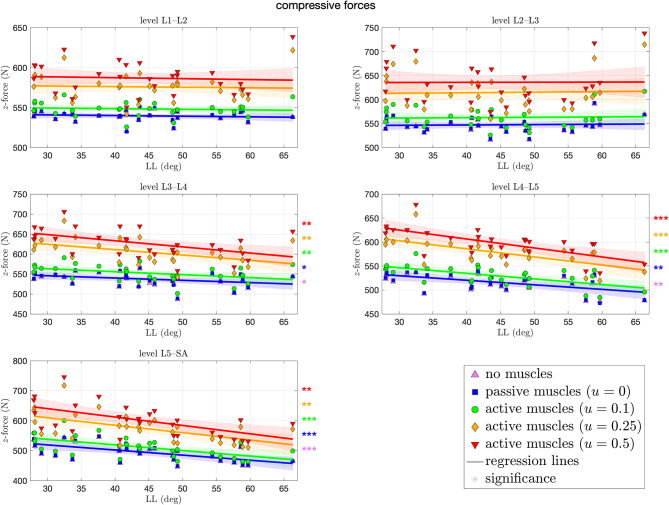
Compressive (*z*-)force between each pair of adjacent vertebrae against lumbar lordosis (LL). Colors and marker symbols of the data points and the corresponding regression lines consistently correspond to the modes: lilac up-pointing triangles for simulations without muscles involved; blue squares for passive muscles; as well as green circles, orange diamonds, and red down-pointing triangles for muscle stimulation of *u* ∈ {0.1, 0.25, 0.5}, respectively. Confidence bands of the regression are shown as pale areas of the corresponding color. The significance of the statistical test is indicated by alongside asterisks (* = tendency with 0.05 ≤ *p* ≤ 0.1, ** = significant with 0.001 ≤ *p* < 0.05, *** = highly significant with *p* < 0.001).

Figure 5 depicts the compressive force resulting at each level of each spinal model at the end of the simulation. These forces ranged from 446–746 N, both at the L5–SA level. For the upper levels L1–L2 and L2–L3, we observed no significant difference across all curvatures. The more caudal the level, the more significant the decrease in force for the cases of no muscles and passive muscles alike, up to −2.8 N (CI: [−4.6, −1] N) per degree LL at the L5–SA level for high muscle stimulation. In case of highly stimulated muscles, the most significant decrease in compressive force happens at the level L4–L5. Tendencies toward an increase in compressive force with LL were not found at all, although the most hyperlordotic spinal model yielded the highest forces in the upper segments. Throughout all levels, an increase in muscle stimulation yielded absolute higher compressive forces.

**Figure 6:**
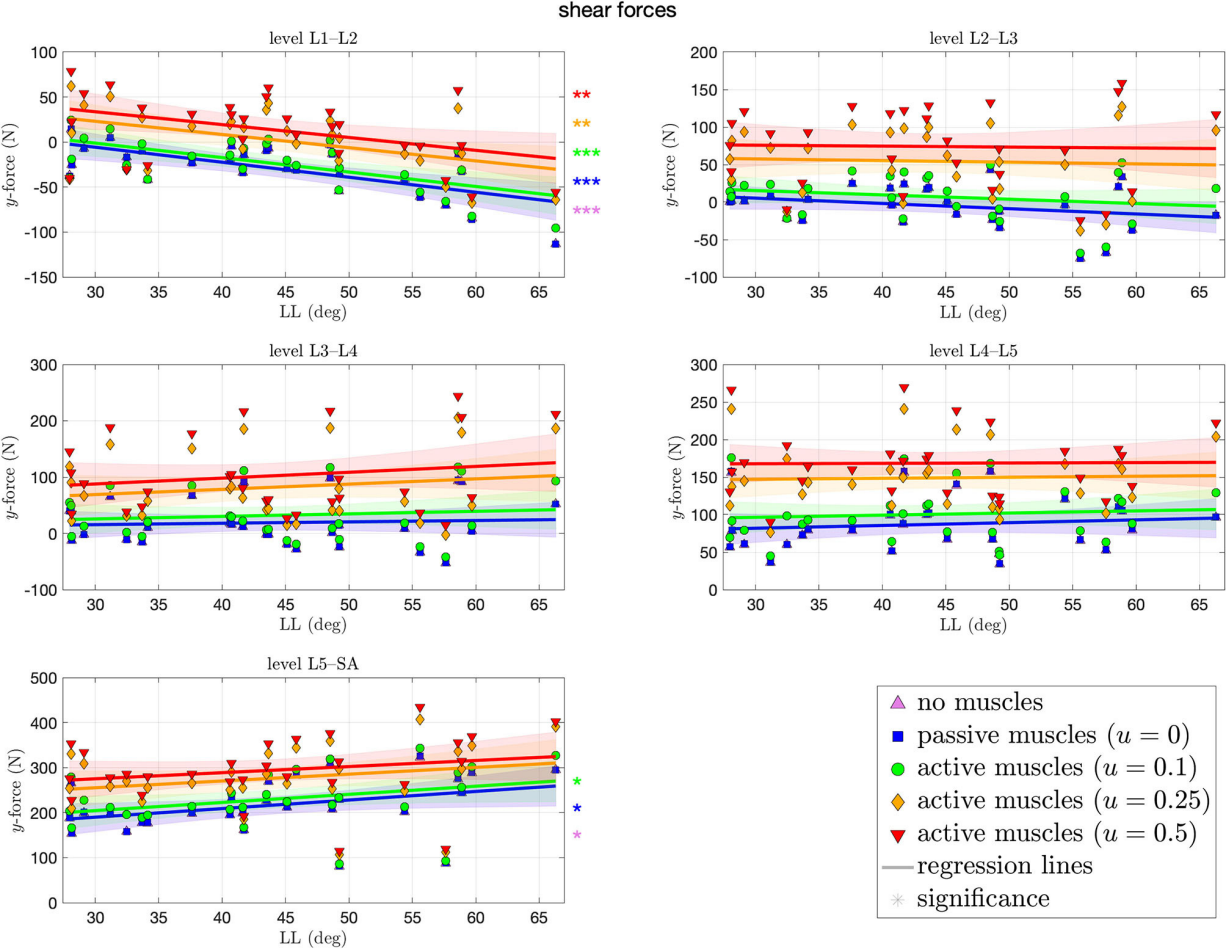
Shear (*y*-)force between each pair of adjacent vertebrae against lumbar lordosis (LL). Colors and marker symbols of the data points and the corresponding regression lines consistently correspond to the modes: lilac up-pointing triangles for simulations without muscles involved; blue squares for passive muscles; as well as green circles, orange diamonds, and red down-pointing triangles for muscle stimulation of *u* ∈ {0.1, 0.25, 0.5}, respectively. Confidence bands of the regression are shown as pale areas of the corresponding color.

Figure 6 depicts the shear forces resulting at each level of each spinal model at the end of the simulation. These forces ranged from −114N (L1–L2) to 438 N (L5–SA). Contrary to the compressive force, shear forces (highly) significantly decreased (increased in posterior direction) with ~1.6 N per degree LL at the L1–L2 level. The more caudal the level, the more of an increase in shear force, absolute and with LL, was observed, although significance is only given on the L5–SA level with up to 1.9 N (CI: [−0.14,4] N) per degree LL. Throughout all levels, an increase in muscle stimulation yielded higher anteriorly directed shear forces.

**Figure 7:**
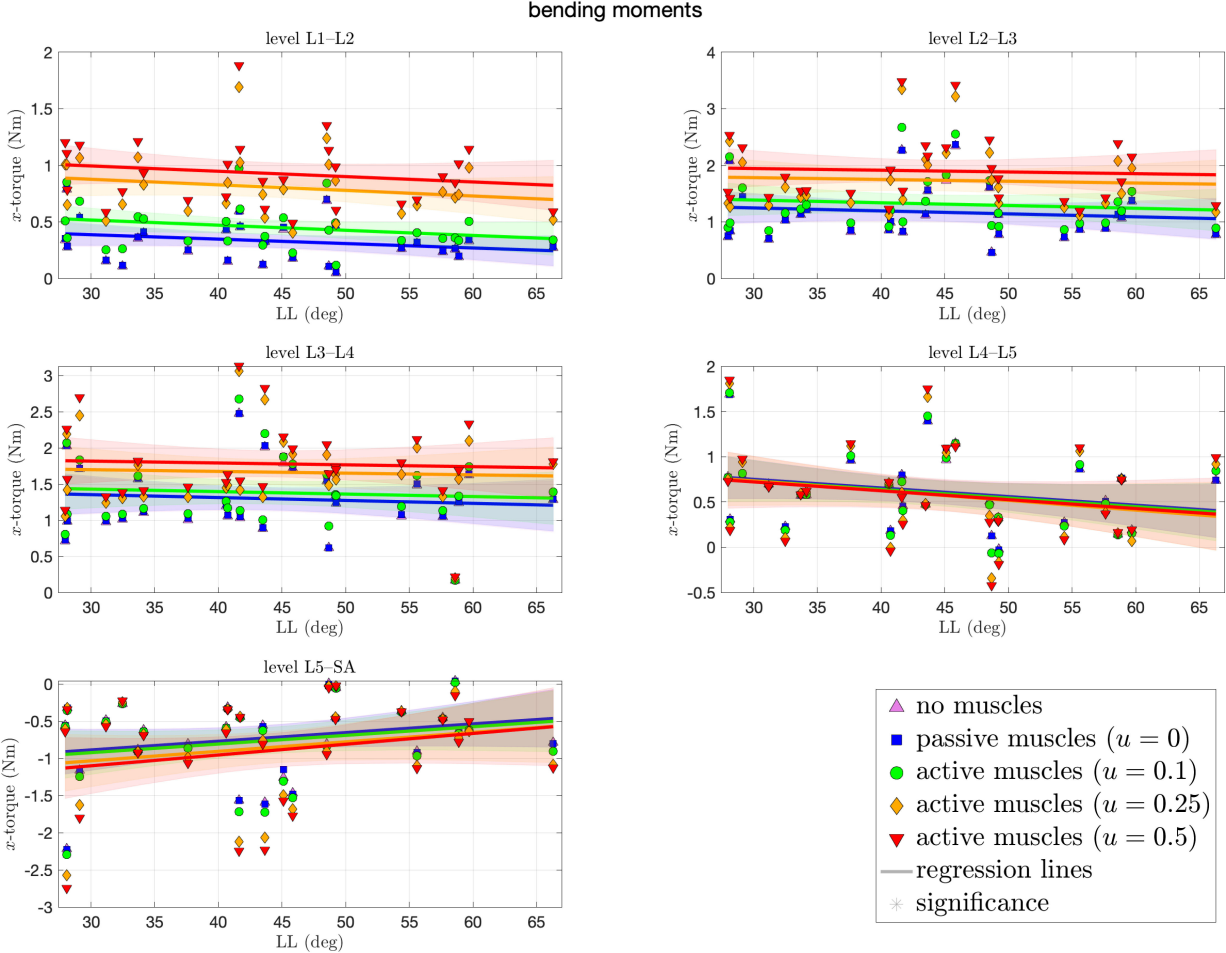
Bending moments (*x*-torques) around the transversal axis between each pair of adjacent vertebrae against lumbar lordosis (LL). Colors and marker symbols of the data points and the corresponding regression lines consistently correspond to the modes: lilac up-pointing triangles for simulations without muscles involved; blue squares for passive muscles; as well as green circles, orange diamonds, and red down-pointing triangles for muscle stimulation of *u* ∈ {0.1, 0.25, 0.5}, respectively. Confidence bands of the regression are shown as pale areas of the corresponding color.

Figure 7 depicts the bending moments around the transversal axis at each level of each spinal model at the end of the simulation. Depending on the degree of muscle stimulation and level, bending moments lie mostly within the range of ±3 Nm, with negative values (indicating forward bending) occur predominantly on the L5–SA level. None of the correlation coefficients was significantly different from zero, i.e., there was no LL dependence. Except for the L4–L5 level, higher muscle stimulation was associated with higher absolute bending moments. Absolute moments around the transversal and longitudinal axes were not significantly different from zero.

**Figure 8:**
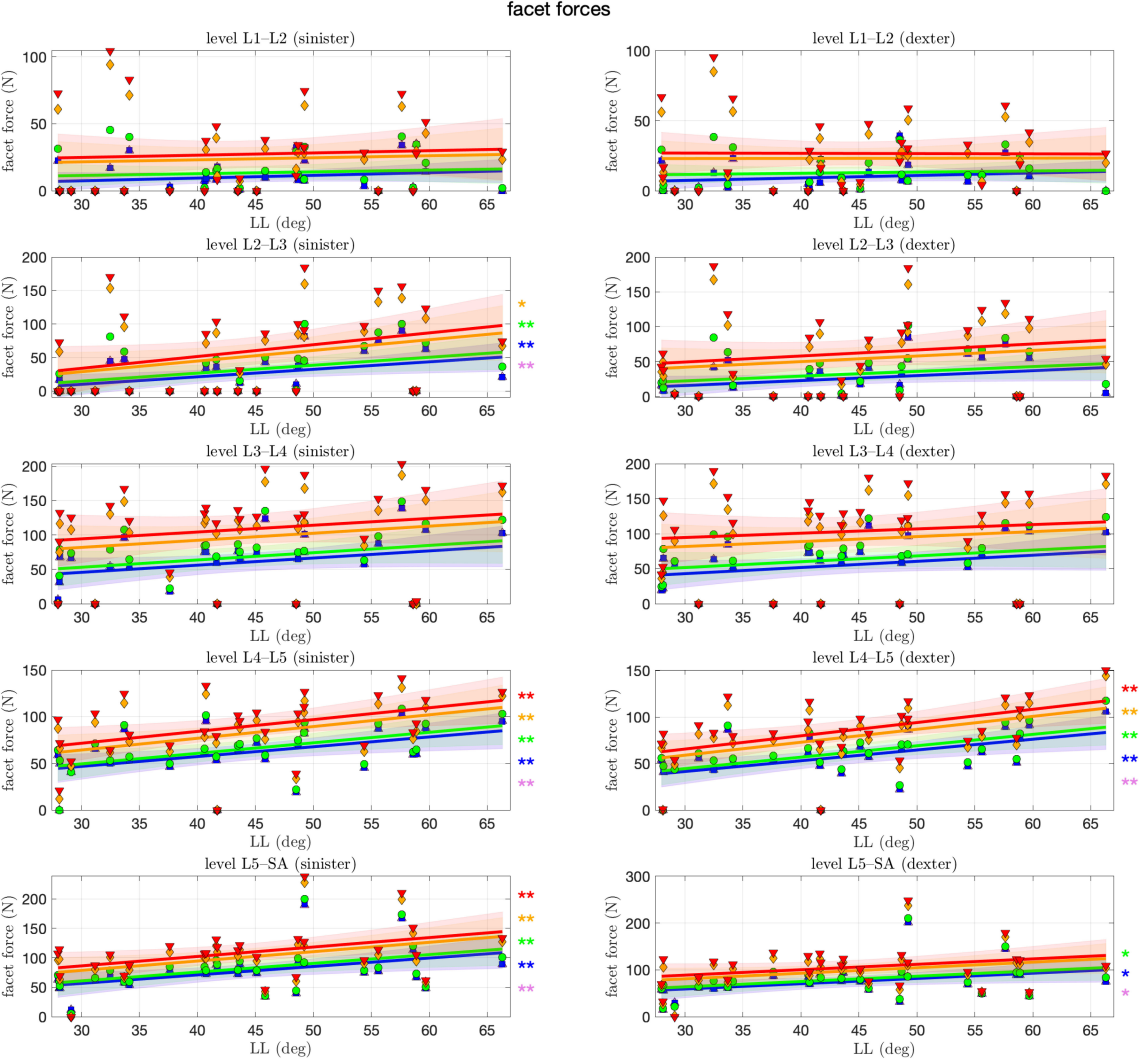
Facet force between each pair of adjacent articular facets against lumbar lordosis (LL). Colors and marker symbols of the data points and the corresponding regression lines consistently correspond to the modes: lilac up-pointing triangles for simulations without muscles involved; blue squares for passive muscles; as well as green circles, orange diamonds, and red down-pointing triangles for muscle stimulation of *u* ∈ {0.1, 0.25, 0.5}, respectively. The significance of the statistical test is indicated by alongside asterisks (* = tendency with 0.05 ≤ *p* ≤ 0.1, ** = significant with 0.001 ≤ *p* < 0.05).

Finally, Figure 8 shows the forces within the sinister (left column of the figure) and dexter (right column) facet joint. On the first glance, we observe in each level and for each mode on each side a trend toward an increase of facet force with LL. This increase is, however, only significant for certain cases, predominantly for the lower levels and lower muscle stimulation, respectively, with an increase of as much as 1.6 N per degree LL at the L5–SA level. Especially on the L1–L2 and L3–L4 level, no significance was found at all. Throughout all levels, an increase in muscle stimulation yielded absolute higher facet forces.

## 4. Discussion

We have shown the effect of varying LL and varying stimulation of the *M. multifidus* on the load distribution within the lumbar spine during forward dynamic compression. Therefore, the CT data from 28 asymptomatic subjects in supine position were transferred into a priorly validated MBS model and underwent standardized loading conditions, representing upright standing. The range of observed LL in our study (28–66.3°) well coincides with prior observations of 28.8–72.9° (Wood et al., 1996, Table 1). Likewise, the changes in LL due to the transition between unloaded supine and loaded standing position consistently account for only a few degrees—cf. Figure 4 (upper left), Wood et al. (1996), and Meakin et al. (2009). Contrary to existing literature on the influence of curvature on spinal loading (Briggs et al., 2007; Bruno et al., 2012, 2017; Galbusera et al., 2014; Naserkhaki et al., 2016), the novelty of our study lies in the formulation of quantitative statements regarding the LL-dependent load distribution during forward dynamic simulations, e.g., “Per one degree increase in LL, the compressive force within the IVD between L5 and SA decreases by 2.8 N (CI: [−4.6, −1] N).” Of course, our absolute output values have to be treated with caution when comparing them directly to *in vivo* (or more elaborated *in silico*) situations. Yet, this study might serve as an impulse for subsequent quantitative corroborations of conjectured coherences. In the following, we consider clinical applications, depending on patient-specific lordosis, and address the role of muscles in the stabilization of the lumbar spine.

### 4.1. Clinical Implications of Varying LL

In clinical practice, physicians are mostly faced with hypolordosis (small LL) due to degenerative diseases, e.g.,reduced height of the intervertebral disk space or flattening of vertebrae due to osteoporotic changes. LL is known to decrease with increasing age (Gelb et al., 1995). With lower LL, the gravity line is located anteriorly, away from its ideal position between the hip joints. As a consequence, compressive force on the intervertebral disks increases (see also our Figure 5), which may favor discopathies that result in further decrease of the LL. A recent meta-analysis of 13 studies with a total of 796 patients (Chun et al., 2017) found that patients with small LL tend to suffer more often from low back pain (independent of the underlying pathology). In a subgroup analysis of five studies, comparing individuals with disk herniation or severe degeneration with a healthy control group, it was further observed that this condition is more likely to occur in individuals with hypolordosis.

Contrary, in a hyperlordotic spine (high LL), the gravity line is located dorsally and thus close to the posterior spinal structures, such as the facet joints and spinous processes. This may favor diseases, such as posterior facets arthritis, Baastrup disease, and spondylolisthesis (Roussouly and Pinheiro-Franco, 2011). Accordingly, we observed in our model a tendency of increasing facet force with increasing LL (see Figure 8). These findings coincide with clinical investigations (Sahin et al., 2015), which found a significant correlation of high LL values with the degree of lumbar facet joint degeneration in CT scans of 723 patients.

Sagittal imbalance in general has been shown to correlate with clinical symptoms (Glassman et al., 2005; Senteler et al., 2014; Rothenfluh et al., 2015). Particularly, an anterior misalignment of C7, and thus of the thoracic weight, results in high compression mainly caused by compensatory muscular forces (Galbusera et al., 2013). This effect is less prominent, yet still identifiable, for backward misalignment (Bassani et al., 2019). Hence, careful evaluation of the individual LL and sagittal profile of patients is of utmost importance to avoid acceleration of degenerative processes. Although lumbar posture can be influenced to a certain extent by muscle hypertrophy training (Scannell and McGill, 2003), in severe cases surgical correction might be required. In general, utilization of subject-specific lumbar spine models might have the ability to assist surgeons to correctly restore the individual balance. These models should be based on (supine) CT data and (standing) radiographs to allow precise measurements of anatomical parameters, such as PI, LL, and other (multi-level) Cobb angles.

### 4.2. The Role of *M. multifidus* in Stabilizing the Lumbar Spine

Lower back muscles, and especially the *M. multifidus*, play an important role in stabilizing the lumbar spine (Macintosh and Bogduk, 1986; Goel et al., 1993; Kaigle et al., 1995; Wilke et al., 1995; Panjabi, 1999; Danneels et al., 2001; Ward et al., 2009). For example, lower back pain patients were shown to have significantly smaller cross-sectional area of their *M. multifidus* (Danneels et al., 2000; Kamaz et al., 2007; Hides et al., 2008) and were less able to voluntarily contract the *M. multifidus* in atrophic segments (Wallwork et al., 2009). This becomes particularly crucial for hypolordotic spines, where holding forces in posterior structures are required. As we have shown in Figure 4, LL increases with increasing muscle force, which is consistent to findings regarding the correlation of muscle volume and LL (Meakin and Aspden, 2012; Meakin et al., 2013).

As we introduced the varying stimulation of the *M. multifidus* as a second ceteris paribus condition in our model, the influence of varying muscle force on the load distribution could be assessed. In Figures 5–8, we observed an increase in compressive and shear forces as well as facet forces with increasing muscle stimulation. However, the significance of the LL dependence of these forces were smaller for higher stimulation values, consistent to observations regarding the application of follower load (Patwardhan et al., 1999). Hence, higher muscle forces seem to compensate for structural deficiencies, see again Scannell and McGill (2003). These findings underline the important interplay between LL and muscle forces (primarily of the *M. multifidus*) in the development of degenerative spinal diseases. Thus, future individualized therapy planning should benefit from careful consideration of the delicate equilibrium of individual curvature and muscle strength.

## 5. Limitations and Perspectives

Several simplifying assumptions regarding our model approach might have an influence on the absolute values of angles, forces, and bending moments that were presented in Figures 4–8. First, our model only consists of pelvis, lumbar vertebrae, IVDs, ligaments, facet joints, and two muscle groups. Yet, geometries and muscle as well as ligament insertion points were extracted from subject-specific CT data. Second, the orientation of the spine with respect to a horizontal L3 endplate in general does not account for real-life variation. As no vertebra C7 was available for most spines, a more realistic balance with respect to the C7 plumb-line could not be performed. Third, as neither data about subject-specific weight or muscle cross-section area was available, loading and muscle forces had to be chosen generically.

For each of the mentioned limitations, it might be worth to conduct a sensitivity analysis regarding the LL dependence of the load distribution. Regarding additional structures and muscles, a quantitative assessment of LL-dependent stabilizing effects could lead to individual muscle hypertrophy training plans toward appropriate posture. Regarding the vertebral orientation, a systematic variation of spinal alignment, as a second independent variable besides LL, might yield configurations with particular high (or low) loading in certain structures that could be connected to lower back pain. Regarding the loading protocol, the herein investigated compression ought to be replaced by common movement tasks, e.g., flexion-extension, equipped with as much individualized information as available.

## 6. Conclusion

The load distribution and stabilizing effect of the *M. multifidus* for different LL were investigated by using simplified forward dynamic MBS models of the lumbar spine. Based on clinical CT data, 28 models with subject-specific geometries, including passive structures as well as two muscle groups, were constructed. To emphasize a possible dependence of load distribution on the LL, standardized orientation and loading conditions as well as generic parameters for passive and active structures were used. Resulting compressive and shear IVD forces, IVD bending moments, and facet forces were displayed and quantitatively connected to LL via the corresponding correlations. Tendentiously, IVD compressive forces in hypolordotic lumbar spines were higher than in hyperlordotic lumbar spines. In contrast, facet joint forces increased with increasing LL. Alterations in shear forces depended on the vertebral level and bending moments did not show any significant change at all. Simulations with higher stimulation of the *M. multifidus* resulted in less significant load distributions, which may be explained by the stabilizing effect of these muscles. The clinical relevance of our findings was discussed.

## Data Availability Statement

The data analyzed in this study is subject to the following licenses/restrictions: medical CT data of patients. Requests to access these datasets should be directed to andreas.mueller@uni-koblenz.de.

## Author Contributions

AM performed the model calculations, modified the MBS model, and drafted the scaffold of the manuscript. RR performed the statistical analysis and supervised the writing. ND developed the basic MBS model components, particularly the geometries on the basis of medical image data. MK and SK equally contributed to the identification of ligament and muscle insertion points as well as the discussion from a medical point of view. AA contributed to the biomechanical discussion and conducted a final review of the manuscript. KG helped in developing the model, provided the MBS graphics, and conducted a final review of the manuscript. All authors contributed to the article and approved the submitted version.

## Conflict of Interest

The authors declare that the research was conducted in the absence of any commercial or financial relationships that could be construed as a potential conflict of interest.
