# Supplementary material for: Load Distribution in the Lumbar Spine During Modeled Compression Depends on Lordosis
Source: Front Bioeng Biotechnol. 2021 Jun 10;9:661258. doi: 10.3389/fbioe.2021.661258 (PMC8222614; doi:10.3389/fbioe.2021.661258)
Supplement: Supplementary file 1 [file Table_1.pdf]

# Supplementary file for the article "Load distribution in the lumbar spine during modeled compression depends on lordosis"

Correspondence*:
Andreas Müller
andreas.mueller@uni-koblenz.de

Table 1: The Pearson correlation coefficients ($r$) and the corresponding $p$-values (in parentheses) of the regression lines in Figs. 4–8 shown with two significant digits.

| quantity / level | L1–L2 $r$ $(p)$ | L2–L3 $r$ $(p)$ | L3–L4 $r$ $(p)$ | L4–L5 $r$ $(p)$ | L5–SA $r$ $(p)$ |
|---|---|---|---|---|---|
| Δ endplate angle | | | | | |
| w/o muscle | 0.63 (0.00033) | 0.7 (3.5e-05) | 0.31 (0.1) | -0.68 (5.7e-05) | -0.65 (0.00017) |
| $u = 0$ | 0.63 (0.00033) | 0.7 (3.7e-05) | 0.31 (0.1) | -0.68 (5.7e-05) | -0.65 (0.00018) |
| $u = 0.1$ | 0.59 (0.00085) | 0.66 (0.00014) | 0.3 (0.12) | -0.64 (0.00023) | -0.63 (0.0003) |
| $u = 0.25$ | 0.53 (0.0037) | 0.56 (0.0018) | 0.2 (0.3) | -0.53 (0.0034) | -0.57 (0.0016) |
| $u = 0.5$ | 0.51 (0.005) | 0.53 (0.0034) | 0.17 (0.39) | -0.5 (0.0062) | -0.54 (0.0031) |
| compressive force | | | | | |
| w/o muscle | -0.12 (0.54) | 0.062 (0.76) | -0.35 (0.07) | -0.55 (0.0024) | -0.62 (0.00036) |
| $u = 0$ | -0.13 (0.52) | 0.05 (0.8) | -0.35 (0.064) | -0.55 (0.0022) | -0.63 (0.00033) |
| $u = 0.1$ | -0.095 (0.63) | 0.039 (0.84) | -0.39 (0.041) | -0.62 (0.0004) | -0.59 (0.00091) |
| $u = 0.25$ | -0.045 (0.82) | 0.033 (0.87) | -0.47 (0.011) | -0.66 (0.00011) | -0.56 (0.0019) |
| $u = 0.5$ | -0.064 (0.75) | 0.0065 (0.97) | -0.49 (0.0082) | -0.69 (4.8e-05) | -0.58 (0.001) |
| shear force | | | | | |
| w/o muscle | -0.65 (0.00015) | -0.27 (0.16) | 0.065 (0.74) | 0.12 (0.56) | 0.35 (0.066) |
| $u = 0$ | -0.65 (0.00015) | -0.28 (0.15) | 0.064 (0.75) | 0.12 (0.56) | 0.35 (0.068) |
| $u = 0.1$ | -0.63 (0.00035) | -0.2 (0.3) | 0.11 (0.59) | 0.085 (0.67) | 0.32 (0.096) |
| $u = 0.25$ | -0.47 (0.011) | -0.052 (0.79) | 0.16 (0.41) | 0.035 (0.86) | 0.24 (0.23) |
| $u = 0.5$ | -0.42 (0.027) | -0.026 (0.9) | 0.16 (0.4) | 0.012 (0.95) | 0.2 (0.3) |
| bending moment | | | | | |
| w/o muscle | -0.24 (0.22) | -0.12 (0.54) | -0.094 (0.63) | -0.27 (0.17) | 0.24 (0.21) |
| $u = 0$ | -0.24 (0.22) | -0.12 (0.55) | -0.091 (0.64) | -0.26 (0.18) | 0.25 (0.21) |
| $u = 0.1$ | -0.25 (0.2) | -0.11 (0.59) | -0.076 (0.7) | -0.25 (0.21) | 0.23 (0.24) |
| $u = 0.25$ | -0.2 (0.31) | -0.061 (0.76) | -0.048 (0.81) | -0.23 (0.24) | 0.21 (0.28) |
| $u = 0.5$ | -0.17 (0.38) | -0.056 (0.78) | -0.05 (0.8) | -0.2 (0.31) | 0.22 (0.25) |
| facet force (sin.) | | | | | |
| w/o muscle | 0.19 (0.33) | 0.43 (0.023) | 0.29 (0.14) | 0.45 (0.016) | 0.44 (0.019) |
| $u = 0$ | 0.19 (0.33) | 0.43 (0.023) | 0.29 (0.14) | 0.45 (0.017) | 0.44 (0.019) |
| $u = 0.1$ | 0.098 (0.62) | 0.37 (0.049) | 0.26 (0.18) | 0.45 (0.015) | 0.44 (0.02) |
| $u = 0.25$ | 0.062 (0.75) | 0.32 (0.1) | 0.19 (0.33) | 0.43 (0.022) | 0.41 (0.032) |
| $u = 0.5$ | 0.06 (0.76) | 0.31 (0.11) | 0.17 (0.4) | 0.41 (0.028) | 0.39 (0.04) |
| facet force (dex.) | | | | | |
| w/o muscle | 0.19 (0.34) | 0.3 (0.12) | 0.27 (0.17) | 0.52 (0.0048) | 0.35 (0.066) |
| $u = 0$ | 0.19 (0.34) | 0.3 (0.12) | 0.27 (0.17) | 0.52 (0.0049) | 0.35 (0.067) |
| $u = 0.1$ | 0.073 (0.71) | 0.24 (0.22) | 0.22 (0.26) | 0.52 (0.0047) | 0.34 (0.076) |
| $u = 0.25$ | 0.0048 (0.98) | 0.17 (0.38) | 0.13 (0.49) | 0.49 (0.0076) | 0.3 (0.12) |
| $u = 0.5$ | -0.0077 (0.97) | 0.16 (0.4) | 0.11 (0.59) | 0.48 (0.0098) | 0.28 (0.15) |

Table 2: Slopes ($s$) and corresponding 95% confidence intervals of the regression lines in Figs. 4–8 shown with two significant digits.

| quantity / level | L1–L2 $s$ [95% CI] | L2–L3 $s$ [95% CI] | L3–L4 $s$ [95% CI] | L4–L5 $s$ [95% CI] | L5–SA $s$ [95% CI] |
|---|---|---|---|---|---|
| $\Delta$ endplate angle | | | | | |
| w/o muscle | 0.044 [0.018,0.071] | 0.038 [0.018,0.059] | 0.0066 [-0.0013,0.015] | -0.031 [-0.048,-0.014] | -0.045 [-0.072,-0.019] |
| $u = 0$ | 0.044 [0.017,0.07] | 0.038 [0.017,0.059] | 0.0066 [-0.0014,0.015] | -0.031 [-0.048,-0.014] | -0.045 [-0.072,-0.019] |
| $u = 0.1$ | 0.043 [0.016,0.071] | 0.038 [0.016,0.059] | 0.0069 [-0.0018,0.016] | -0.03 [-0.048,-0.012] | -0.046 [-0.073,-0.018] |
| $u = 0.25$ | 0.044 [0.013,0.076] | 0.036 [0.012,0.061] | 0.0062 [-0.0054,0.018] | -0.029 [-0.05,-0.0086] | -0.046 [-0.077,-0.015] |
| $u = 0.5$ | 0.044 [0.012,0.076] | 0.035 [0.01,0.06] | 0.0058 [-0.0071,0.019] | -0.028 [-0.05,-0.0071] | -0.046 [-0.078,-0.014] |
| compressive force | | | | | |
| w/o muscle | -0.075 [-0.31,0.16] | 0.09 [-0.46,0.64] | -0.55 [-1.2,0.05] | -0.91 [-1.5,-0.28] | -1.7 [-2.7,-0.67] |
| $u = 0$ | -0.08 [-0.32,0.16] | 0.073 [-0.48,0.63] | -0.57 [-1.2,0.039] | -0.95 [-1.6,-0.3] | -1.7 [-2.7,-0.68] |
| $u = 0.1$ | -0.078 [-0.38,0.23] | 0.077 [-0.68,0.83] | -0.74 [-1.5,-0.018] | -1.2 [-1.9,-0.46] | -1.9 [-3.1,-0.67] |
| $u = 0.25$ | -0.071 [-0.66,0.52] | 0.12 [-1.2,1.4] | -1.3 [-2.4,-0.27] | -1.7 [-2.6,-0.73] | -2.5 [-4.2,-0.81] |
| $u = 0.5$ | -0.12 [-0.81,0.58] | 0.025 [-1.5,1.5] | -1.5 [-2.7,-0.35] | -1.9 [-2.9,-0.84] | -2.8 [-4.6,-0.99] |
| shear force | | | | | |
| w/o muscle | -1.7 [-2.6,-0.7] | -0.69 [-1.6,0.26] | 0.25 [-1.2,1.7] | 0.37 [-0.84,1.6] | 1.9 [-0.14,4] |
| $u = 0$ | -1.7 [-2.6,-0.7] | -0.7 [-1.7,0.26] | 0.25 [-1.2,1.7] | 0.37 [-0.84,1.6] | 1.9 [-0.15,4] |
| $u = 0.1$ | -1.6 [-2.6,-0.64] | -0.58 [-1.7,0.5] | 0.45 [-1.2,2.1] | 0.29 [-0.98,1.5] | 1.8 [-0.32,3.9] |
| $u = 0.25$ | -1.5 [-2.6,-0.29] | -0.22 [-1.8,1.4] | 0.93 [-1.2,3.1] | 0.14 [-1.3,1.6] | 1.5 [-0.91,3.9] |
| $u = 0.5$ | -1.4 [-2.7,-0.14] | -0.12 [-1.9,1.7] | 1 [-1.3,3.4] | 0.05 [-1.5,1.6] | 1.4 [-1.2,3.9] |
| bending moment | | | | | |
| w/o muscle | -0.0039 [-0.01,0.0023] | -0.0053 [-0.022,0.011] | -0.0041 [-0.021,0.012] | -0.0095 [-0.023,0.004] | 0.012 [-0.0064,0.03] |
| $u = 0$ | -0.0039 [-0.01,0.0023] | -0.0052 [-0.022,0.011] | -0.004 [-0.021,0.013] | -0.0095 [-0.023,0.0043] | 0.012 [-0.0063,0.03] |
| $u = 0.1$ | -0.0045 [-0.011,0.0023] | -0.0048 [-0.022,0.012] | -0.0034 [-0.02,0.013] | -0.0095 [-0.024,0.0052] | 0.012 [-0.0074,0.031] |
| $u = 0.25$ | -0.0049 [-0.014,0.0045] | -0.0032 [-0.023,0.017] | -0.0024 [-0.021,0.016] | -0.011 [-0.028,0.0068] | 0.013 [-0.0099,0.035] |
| $u = 0.5$ | -0.0047 [-0.015,0.0055] | -0.003 [-0.024,0.018] | -0.0026 [-0.022,0.017] | -0.0098 [-0.028,0.0087] | 0.015 [-0.0099,0.039] |
| facet force (sin.) | | | | | |
| w/o muscle | 0.2 [-0.2,0.6] | 1.1 [0.13,2.1] | 1 [-0.32,2.4] | 1.1 [0.17,1.9] | 1.4 [0.21,2.7] |
| $u = 0$ | 0.2 [-0.2,0.61] | 1.1 [0.13,2.1] | 1 [-0.33,2.4] | 1.1 [0.16,1.9] | 1.4 [0.2,2.7] |
| $u = 0.1$ | 0.13 [-0.38,0.65] | 1.2 [-0.01,2.4] | 1.1 [-0.46,2.6] | 1.1 [0.19,2.1] | 1.5 [0.2,2.8] |
| $u = 0.25$ | 0.15 [-0.76,1.1] | 1.6 [-0.3,3.5] | 1 [-1,3.1] | 1.3 [0.15,2.4] | 1.6 [0.11,3.1] |
| $u = 0.5$ | 0.17 [-0.89,1.2] | 1.8 [-0.39,3.9] | 0.99 [-1.3,3.2] | 1.3 [0.11,2.4] | 1.6 [0.047,3.2] |
| facet force (dex.) | | | | | |
| w/o muscle | 0.18 [-0.18,0.53] | 0.71 [-0.17,1.6] | 0.88 [-0.36,2.1] | 1.2 [0.31,2] | 1.1 [-0.082,2.3] |
| $u = 0$ | 0.18 [-0.18,0.54] | 0.71 [-0.18,1.6] | 0.88 [-0.37,2.1] | 1.2 [0.31,2] | 1.1 [-0.085,2.3] |
| $u = 0.1$ | 0.083 [-0.35,0.52] | 0.68 [-0.39,1.8] | 0.83 [-0.58,2.2] | 1.2 [0.33,2.1] | 1.1 [-0.13,2.4] |
| $u = 0.25$ | 0.0097 [-0.74,0.76] | 0.8 [-0.94,2.5] | 0.71 [-1.3,2.7] | 1.4 [0.32,2.5] | 1.2 [-0.31,2.7] |
| $u = 0.5$ | -0.018 [-0.9,0.86] | 0.86 [-1.1,2.8] | 0.61 [-1.6,2.8] | 1.4 [0.3,2.5] | 1.2 [-0.43,2.7] |
